# Why is high-risk drinking more prevalent among men than women? evidence from South Korea

**DOI:** 10.1186/1471-2458-12-101

**Published:** 2012-02-06

**Authors:** Woojin Chung, Seungji Lim, Sunmi Lee

**Affiliations:** 1 Department of Health Policy and Management, Graduate School of Public Health, Yonsei University, Seoul, South Korea; 2 Institute of Health Services Research, Yonsei University, Seoul, South Korea; 3 Department of Public Health, Yonsei University Graduate School, Seoul, South Korea; 4 Health Insurance Policy Research Institute, National Health Insurance Corporation, Seoul, South Korea

## Abstract

**Background:**

It is important to identify and quantify the factors that affect gender differences in high-risk drinking (HRD), from both an academic and a policy perspective. However, little is currently known about them. This study examines these factors and estimates the percentage contribution each makes to gender differences in HRD.

**Methods:**

This study analyzed information on 23,587 adults obtained from the Korea National Health and Nutrition Surveys of 1998, 2001, and 2005. It found that the prevalence of HRD was about 5 times higher among men (0.37) than women (0.08). Using a decomposition approach extended from the Oaxaca-Blinder method, we decomposed the gender difference in HRD to an "overall composition effect" (contributions due to gender differences in the distribution of observed socio-economic characteristics), and an "overall HRD-tendency effect" (contributions due to gender differences in tendencies in HRD for individuals who share socio-economic characteristics).

**Results:**

The HRD-tendency effect accounted for 96% of the gender difference in HRD in South Korea, whereas gender differences in observed socio-economic characteristics explained just 4% of the difference. Notably, the gender-specific HRD-tendency effect accounts for 90% of the gender difference in HRD.

**Conclusion:**

We came to a finding that gender-specific HRD tendency is the greatest contributor to gender differences in HRD. Therefore, to effective reduce HRD, it will be necessary to understand gender differences in socioeconomic characteristics between men and women but also take notice of such differences in sociocultural settings as they experience. And it will be also required to prepare any gender-differentiated intervention strategy for men and women.

## Background

Alcohol drinking is recognized as a leading risk factor in disease, injury and death worldwide. Additionally, men and women show different alcohol drinking behaviour, and numerous studies have reported that men are more likely to consume greater amounts of alcohol and cause more problems by doing so than women [1]. However, the magnitude of these gender differences varies from country to country and the difference has persisted over time, both in European countries [2-5] and many developing countries [6].

Why do gender differences occur in alcohol drinking behaviour? Previous studies reported that gender differences in alcohol drinking behaviour were associated with physiological or biological predispositions [7-12] and gender-role orientations [13-17]. In particular, the social norms of South Korean ("Korea" hereafter) are still dominated primarily by a traditional values based on Confucianism, so social activities and alcohol drinking culture were permitted only to adult men in past. Thus, it was reported that these traditional sociocultural norms of Korea are associated with gender differences in our contemporary alcohol drinking behavior [16,17]. Likewise, in case of Russia that is a country with high alcohol drinking rate worldwide and faces high burden of resulting diseases, it was reported that social norms proscribing women from drinking alcohol which was considered as appropriate masculine behaviour, and women's social roles such as caring for their families, were associated with gender differences in alcohol drinking behavior [18-20]. In addition, it was reported that both social norms and women's social roles of Russia were contributors to the greatest increase in men mortality and the highest men/women differences in life expectancy around the world [18,21].

Meanwhile, other studies have concluded that, regardless of gender, a person's socio-economic characteristics are closely associated with men/women alcohol drinking behaviour. Most previous studies indicated that excessive alcohol drinking decreased with age or related to the inverted U-shape in both men and women [18,22-24], although there were differences in the significance. In general, alcohol drinking is higher in unmarried persons [18,25,26]. By gender, a men's marital status made little difference to his drinking behaviour, while a women's excessive alcohol drinking increased when she was single, separated, divorced, or widowed [22,23]. The relationship between education level and drinking behaviour was mixed. In some studies, a lower level of education led to greater alcohol drinking in both men and women [22,27-30]. However, some studies have suggested that better educated women are more likely to abuse alcohol than their less well educated counterparts [31]. The relationship between occupation and drinking behavior was unclear, but many studies found that alcohol drinking increased with unemployment [2,32-34]. By income level, alcohol drinking in both men and women was most likely to rise when there were economic difficulties [22,23,35]. The relationship between obesity and drinking behavior was unclear, although excessive alcohol drinking led to a higher body mass index (BMI), or a J-shaped relationship was observed [36-38]. Heavier stress levels correlated with greater alcohol drinking [18,29].

The per capita pure alcohol consumption of Korea adults of 15 years or older amounted to 19.9 l per annum, which stands at significantly higher level than worldwide average (6.1 l) and Western Pacific region's average (6.2 l) [39]. Accordingly, Korea's socio-economic cost of alcohol drinking, which includes medical care utilization, productivity reduction, premature death, property loss and administration processing, accounts for 2.9% of Korea's GDP [40]. This indicates that Korean people suffer from the very serious adverse effects of alcohol drinking in comparison with other countries [40]. It is estimated that such an excessive alcohol drinking and the resulting adverse effects are possibly attributed to Korean culture, which is very generous to alcohol drinking. For Korean men, it is notable that alcohol is a social lubricant, and alcohol consumption is considered essential to many business and social gatherings. Conversely, Korean women very rarely joined social activities and were excluded from the men-driven culture of alcohol drinking, because they were traditionally expected to rear and take care of children at home, and help husbands and parents-in-law. Lately, however, there has been a rapidly increasing number of Korean women who have joined business and social activities, and young Korean women take a more positive part in social activities for their economic causes and self-realization, so there is a rapidly increasing rate of alcohol consumption in recent years than before. Although the proportion of men that engage in excessive alcohol drinking is usually much higher than that of women, Korea has undergone a notable transition in the incidence of drinking in men versus women. In practice, the prevalence of drinking (the rate of drinkers who drank alcohol once or more times during the last month) in Korean adult men at age of 20 or older increased from 74.8% in 1992 to 76.3% in 2005, whereas it rose noticeably from 21.7 to 40.8% in Korean adult women of the same age group [41].

In this study, high-risk drinking ("HRD" hereafter) means the level of excessive alcohol drinking which is likely to cause chronic problems (e.g. cancers, alcoholic psychosis and dependence, cardiovascular diseases, etc.), as well as acute problems (e.g. accidental fall, fire, drowning, industrial accidents, violence, etc.). Many prior studies propose an international cut-off of HRD resulting in these acute and chronic problems, viz. pure alcohol consumption over 60 g per drinking day (men) and over 40 g per drinking day (women) [42-44]. However, there is little definite information on the relationship between socio-economic characteristics and HRD in Korea or on gender differences. Thus, it is important to identify and quantify factors that affect gender differences in HRD in Korea, from both an academic and a policy perspective. In this study, we examined these factors and estimate the percentage contribution each makes to gender differences in HRD.

## Methods

### Data source and study sample

We have analyzed data collected from the Korea National Health and Nutrition Examinations Surveys ("KNHNES" hereafter) of 1998, 2001, and 2005 [41,45,46]. KNHNES is the largest-scale nationwide survey of all health-related surveys conducted in Korea. The first survey of KNHNES was conducted in 1998, and has subsequently been conducted every 3 years. It consists of a health examination, health and nutrition behaviour questionnaire survey, which are conducted by interviewers who visit interviewees on site; and it also identifies useful information on socio-economics, demographics, health and lifestyle factors. All findings produced from this survey were used as reference materials with a view to setting and assessing new goals for better national health and developing effective health promotion programs. Furthermore, the results contribute to ensuring that Korea, an OECD member country, provides comparable statistic data for the international society. Some 4,395 households in 1998, 4,400 in 2001, and 4,600 in 2005 were randomly sampled from all prefectures. The respective response rates were 95.2, 88.5, and 92.7%. We analyzed 23,587 individuals aged 20 years or over (10,796 men and 12,791 women) who provided information on drinking and characteristics potentially related to drinking. We received approval from the Institutional Review Board to review, analyze, and report on these data.

### Measures and variables

Our dependent variable is whether a person engages in HRD. In this study, HRD means the level of excessive alcohol drinking which is likely to cause chronic problems (e.g. cancers, alcoholic psychosis and dependence, cardiovascular diseases, cerebral apoplexy, gastritis and pancreatitis, etc.), as well as acute problems (e.g. accidental fall, fire, drowning, industrial accidents, suicide, violence, child abuse, etc.). Many prior studies, including reports by WHO, propose an international cut-off of HRD resulting in these acute and chronic problems, viz. pure alcohol consumption over 60 g per drinking day (men) and over 40 g per drinking day (women) [42-44]. Thus, HRD in this study was defined as drinking > 60 g pure alcohol per drinking day for men and > 40 g for women. Therefore, the level of HRD in Korea amounts to over 5 cups of beer (> 330 ml × 5) or 8 cups of soju (> 50 ml × 8) per drinking day for men and also amounts to over 3 cups of beer (> 330 ml × 3) or 5 cups of soju (> 50 ml × 5) per drinking day for women. We calculated pure alcohol consumption as recommended by WHO: Pure alcohol consumption = alcohol consumption per drinking day (ml) × alcohol concentration by type of beverage (%) × specific gravity of alcohol (0.79 g/ml ethanol).

Explanatory variables for the analysis included age, marital status, education level, occupational status, household income, body mass index (BMI), stress level and survey year. The variables that turned out to be examinable in the Korea National Health and Nutrition Examinations Surveys were selected as independent variables among the variables that were linked to HRD in previous studies. Individuals were divided into 5 age groups: < 30, 30-39, 40-49, 50-59, and 60+ years. They were also grouped according to marital status: married and non-married (never married, separated, widowed or divorced). Education level was divided into lower than college level and college level or higher. Occupational status was classified into 3 groups: non-manual labor, manual labor, and unemployed. Non-manual labour included managers, professionals, technicians and clerks, whereas manual labour included service and sales workers, agricultural and fishery workers, crafts and related trade workers, plant and machine operators and assemblers and elementary occupations. Those who were not in the labour market (retired, students and housewives) were categorized as unemployed. To adjust for household size, the monthly household income was divided by the square root of the household size [47] and categorized into quartiles according to information on the distribution of the income at each survey year. BMI was categorized into 4 groups: < 20 (underweight), 20-24 (normal weight), 25-29 (over-weight), and 30+ (obese). Stress level was divided into 4 groups. The subjects were asked how much stress they usually suffered in daily routine life and they were asked to choose their answer from among the 4 choices given: very low, low, high, and very high. Variables indicating survey year were included to capture the effect of any time trend.

### Normalized, detailed decomposition approach

To decompose factors underlying gender differences in HRD and to obtain the separate contributions of factors we used a normalized, detailed decomposition approach [48], extended from the Oaxaca-Blinder method [49,50]. This approach overcame both the "path dependence" problem of non-linear models, in which the independent contribution of one variable to a different variable depends on the values of the other variables, on the order in which these variables are entered in the decomposition, and on the "identification" problem in which the contributions of sets of dummy variables vary with the choice of reference groups [48].

In the decomposition process, we decomposed the gender difference in HRD into an "overall composition effect" and an "overall HRD-tendency effect." The overall composition effect denotes contributions due to gender differences in the distribution of observed socio-economic characteristics. The overall HRD-tendency effect was again decomposed into a "pure HRD-tendency effect" and a "gender-specific HRD-tendency effect." A pure HRD-tendency effect denotes contributions associated with gender differences in HRD-tendency due solely to socio-economic characteristics. A gender-specific HRD-tendency effect represents contributions due to gender differences in gender-specific constant terms. Thus, it represents a component influencing HRD-tendency that cannot be attributed to gender differences in socio-economic characteristics or pure tendencies. The decomposition approach used here is summarized as follows.

We assume that there are N (indexed, i = 1 ... N) persons in 2 mutually exclusive and collectively exhaustive groups, k = 1 (men) or 2 (women), each group containing Nk persons. We define the variable $S_{i}^{k}$ as $S_{i}^{k}=1$, if person i of group k engages in HRD and $S_{i}^{k}=0$, if (s)he does not. When a multivariate logistic model is used, the predicted probability of a person engaging in HRD is

$$\mathrm{Pr}\left(S_{i}=1\right)=\frac{\exp\left(X_{i}^{k}{\hat{\beta }}^{k}\right)}{1+\exp\left(X_{i}^{k}{\hat{\beta }}^{k}\right)}=F\left(X_{i}^{k}{\hat{\beta }}^{k}\right)$$

where $X_{i}^{k}=\left\{\left(X_{ij},\;\text{j}=1\ldots \text{J}\right)\right)$ represents the vector of observed characteristics, for the person, on J variables, which determine the probability of a person engaging in HRD, and ${\hat{\beta }}^{k}=\left\{\left(\beta_{j}^{k},\;\text{j}=1\ldots \text{J}\right)\right)$ is the associated vector of coefficient estimates for the person. The average probability of persons from group k engaging in HRD is

$$\bar{P}\left(X_{i}^{k},{\hat{\beta }}^{k}\right)=N_{k}^{-1}\overset{N_{k}}{\underset{i=1}{\sum }}F\left(X_{i}^{k}{\hat{\beta }}^{k}\right)$$

Here, the difference in the observed proportion of persons engaging in HRD between the two groups $\left({\bar{S}}_{k}\right)$ is equal to the difference in the average predicted probability from models estimated for the 2 groups, or

(1) $${\bar{S}}_{1}-{\bar{S}}_{2}=\bar{P}\left(X_{i}^{1},{\hat{\beta }}^{1}\right)-\bar{P}\left(X_{i}^{2},{\hat{\beta }}^{2}\right)$$

If we need to decompose the overall difference into components that reflect the differences in population composition and the differences in tendency (coefficients) between the 2 groups, Eq. (1) can be rewritten as

(2) $${\bar{S}}_{1}-{\bar{S}}_{2}=\left\{\bar{P}\left(X_{i}^{1},{\hat{\beta }}^{1}\right)-\bar{P}\left(X_{i}^{2},{\hat{\beta }}^{1}\right)\right\}+\left\{\bar{P}\left(X_{i}^{2},{\hat{\beta }}^{1}\right)-\bar{P}\left(X_{i}^{2},{\hat{\beta }}^{2}\right)\right\}$$

The first term appearing in the sum in Eq. (2) is the portion of the differential attributed to differences in population composition, which is the predicted probability of engaging in HRD of Group 1 minus the predicted probability of Group 2, if Group 2 faces the same tendency as Group 1. This component reflects the contribution to differences that would have occurred if the 2 groups differed only with respect to population composition. The second term in Eq. (2) is the portion of the differential attributed to differences in tendency, which assesses a change in HRD that would have occurred if group characteristics were held fixed at the levels of Group 2. To obtain the contribution of each independent characteristic on the component of the difference in a person engaging in HRD, we partitioned the portion of the differential attributed to changes in population composition and the portion of the differential due to changes in tendency into components that reflect the unique contribution of the math covariate by a weighting component. The weighting component for the first portion is

$$W_{\Delta x_{j}}=\frac{\left({\bar{x}}_{1_{j}}-{\bar{x}}_{2_{j}}\right)b_{1_{j}}}{\sum_{j=1}^{J}\left({\bar{x}}_{1_{j}}-{\bar{x}}_{2_{j}}\right)b_{1_{j}}}$$

and that for the second portion is

$$W_{\Delta b_{j}}=\frac{{\bar{x}}_{2_{j}}\left(b_{1_{j}}-b_{2_{j}}\right)}{\sum_{j=1}^{J}{\bar{x}}_{2_{j}}\left(b_{1_{j}}-b_{2_{j}}\right)}$$

$$\text{here}\;\sum_{j=1}^{J}W_{\Delta x_{j}}=\sum_{j=1}^{J}W_{\Delta b_{j}}=1.$$

Because the estimates do not provide information concerning the precision of the contributions to the difference in persons engaging in HRD, we derived the standard errors of the detailed contributions for each component and conducted significance testing using the delta method [51].

### Analytic procedures

First, we tested gender differences in the observed socio-economic characteristics (composition) and gender differences for the prevalence of HRD using a *t*-test. Second, multivariate logistic regression analyses were used to calculate the adjusted odds ratio with 95% confidence intervals for the association between characteristics and HRD of subjects. This was done separately for men and women using the same variables for easy comparison. Third, potential multi-collinearity was checked and discarded independent variables that resulted in values of the variance inflation factor > 2.5 from each multivariate logistic regression model [52]. Finally, to decompose the main factors underlying gender differences in HRD and obtain the separate contributions of each factor, the normalized detailed decomposition approach mentioned previously was used.

## Results

Table 1 shows the gender differences in socio-economic characteristics and prevalence of HRD. Regarding characteristic composition, compared with women, men had a significantly higher proportion in persons aged 30-49 years, married, with a college or higher level of education, having a job, belonging to the highest and second highest quartile group of household income, and having a BMI below 20.

**Table 1:** Descriptive statistics (n = 23587)

	Composition (%)				High-risk Drinking (%)			
	**Males**	**Females**	**Difference**	***p*-value**	**Males**	**Females**	**Difference**	***p*-value**

All	-	-	-	-	0.374	0.076	0.298	< 0.0001

Age (years)								

< 30	0.167	0.175	-0.008	0.1100	0.418	0.179	0.239	< 0.0001

30-39	0.251	0.235	0.016	0.0057	0.431	0.077	0.355	< 0.0001

40-49	0.243	0.221	0.022	< 0.0001	0.423	0.076	0.347	< 0.0001

50-59	0.157	0.149	0.007	0.1154	0.343	0.044	0.300	< 0.0001

≥ 60	0.184	0.221	-0.037	< 0.0001	0.218	0.017	0.201	< 0.0001

Marital status								

Non-married	0.249	0.320	-0.071	< 0.0001	0.415	0.121	0.294	< 0.0001

Married	0.751	0.680	0.071	< 0.0001	0.360	0.055	0.305	< 0.0001

Education								

Lower than college	0.648	0.774	-0.127	< 0.0001	0.359	0.066	0.293	< 0.0001

College or higher	0.352	0.226	0.127	< 0.0001	0.400	0.111	0.290	< 0.0001

Occupational status								

Unemployed	0.222	0.537	-0.316	< 0.0001	0.316	0.059	0.257	< 0.0001

Non-manual occupation	0.233	0.124	0.109	< 0.0001	0.398	0.132	0.266	< 0.0001

Manual occupation	0.546	0.339	0.207	< 0.0001	0.387	0.083	0.304	< 0.0001

Household income								

Lowest quartile	0.214	0.251	-0.036	< 0.0001	0.319	0.057	0.262	< 0.0001

2nd lowest quartile	0.259	0.255	0.004	0.4481	0.363	0.080	0.283	< 0.0001

2nd highest quartile	0.262	0.248	0.014	0.0112	0.403	0.088	0.315	< 0.0001

Highest quartile	0.264	0.247	0.018	0.0021	0.401	0.080	0.321	< 0.0001

Body mass index								

< 20	0.349	0.313	0.036	< 0.0001	0.386	0.114	0.273	< 0.0001

20-24	0.425	0.454	-0.029	< 0.0001	0.337	0.056	0.281	< 0.0001

25-29	0.208	0.206	0.002	0.6573	0.418	0.059	0.360	< 0.0001

≥ 30	0.018	0.027	-0.009	< 0.0001	0.492	0.106	0.386	< 0.0001

Stress								

Very low	0.157	0.162	-0.006	0.2300	0.305	0.046	0.259	< 0.0001

Low	0.488	0.485	0.003	0.6490	0.367	0.073	0.294	< 0.0001

High	0.291	0.289	0.001	0.8484	0.409	0.092	0.316	< 0.0001

Very high	0.065	0.063	0.002	0.6074	0.434	0.104	0.330	< 0.0001

Survey year								

1998	0.367	0.356	0.012	0.0626	0.343	0.029	0.314	< 0.0001

2001	0.313	0.313	0.000	0.9966	0.354	0.072	0.282	< 0.0001

2005	0.320	0.332	-0.012	0.0571	0.429	0.131	0.298	< 0.0001

*Notes*: Standard deviations are in parentheses; a non-married marital status includes never-married, separated, widowed, and divorced participants; unemployed includes students and housewives; household income is standardized by household size; and *p*-values for mean differences are based on the *t*-test.

The proportion of HRD was about 5 times higher in men (0.374) than women (0.076), and the gender difference in HRD was 0.298. In every socio-economic category, the proportion of HRD was much higher in men than women, and significant gender differences were found between them. In particular, the largest gender difference in HRD was in persons having a BMI > 30 (0.386), whereas the smallest gender difference was seen in persons aged 60 years or over (0.201).

Table 2 shows the adjusted odds ratio of HRD and its 95% confidence interval in men and women. In men, age had a negative association with HRD, but the pattern was much clearer in women than in men. Being married decreased the likelihood of HRD more conspicuously in women than men. A higher level of education significantly reduced the likelihood of HRD in women, but was not significant in men. Having a manual labour job had a positive impact on engaging in HRD, but this was stronger in women than men. The level of income was significantly and positively associated with HRD in men, but not in women. The pattern of association of BMI with HRD was irregular, but was approximately U-shaped. A higher level of stress was positively associated with HRD, but the positive association was stronger in women than men. During the period from 1998 to 2005, the likelihood of HRD rose in men and women, but the increase was faster in women than men. However, Table 2 shows differences between men and women in the odds ratio and the significance of socio-economic factors to explain their high-risk drinking. This finding may partially explain differences in HRD between men and women. On the other hand, it may support the fact that socio-economic factors and their associated likelihood of HRD may have limitations in explaining the difference in HRD between men and women.

**Table 2:** Factors influencing high-risk drinking (n = 23587)

	Males (n = 10796)			Females (n = 12791)		
	**OR**	**95% CI**	***p*-value**	**OR**	**95% CI**	***p*-value**

Age (years)						

< 30	1.000			1.000		

30-39	1.034	0.893-1.197	0.6566	0.513	0.416-0.634	< 0.0001

40-49	0.978	0.834-1.145	0.7795	0.405	0.322-0.509	< 0.0001

50-59	0.705	0.594-0.838	< 0.0001	0.195	0.146-0.260	< 0.0001

≥ 60	0.412	0.344-0.493	< 0.0001	0.060	0.043-0.085	< 0.0001

Marital status						

Non-married	1.000			1.000		

Married	0.872	0.773-0.984	0.0266	0.461	0.386-0.550	< 0.0001

Education						

Lower than college	1.000			1.000		

College or higher	0.949	0.855-1.052	0.3192	0.667	0.555-0.801	< 0.0001

Occupation						

Unemployed	1.000			1.000		

Non-manual	1.049	0.907-1.213	0.5206	1.030	0.839-1.265	0.7749

Manual	1.176	1.043-1.326	0.0083	1.380	1.167-1.632	0.0002

Household income						

Lowest quartile	1.000			1.000		

2nd lowest quartile	1.005	0.888-1.137	0.9379	1.137	0.920-1.406	0.2336

2nd highest quartile	1.136	1.001-1.289	0.0479	1.165	0.941-1.442	0.1605

Highest quartile	1.143	1.002-1.304	0.0464	1.138	0.910-1.423	0.2557

Body mass index						

< 20	1.000			1.000		

20-24	0.911	0.830-0.999	0.0488	0.769	0.655-0.903	0.0014

25-29	1.238	1.109-1.383	0.0002	1.062	0.860-1.311	0.5779

≥ 30	1.495	1.113-2.007	0.0075	1.698	1.156-2.495	0.0070

Stress						

Very low	1.000			1.000		

Low	1.024	0.905-1.159	0.7064	1.023	0.806-1.298	0.8545

High	1.230	1.078-1.404	0.0021	1.385	1.082-1.772	0.0097

Very high	1.408	1.167-1.700	0.0004	1.782	1.285-2.471	0.0005

Survey year						

1998	1.000			1.000		

2001	1.046	0.947-1.155	0.3720	2.686	2.162-3.337	< 0.0001

2005	1.488	1.349-1.642	< 0.0001	5.781	4.711-7.095	< 0.0001

Log likelihood		-6896			-2910	

C-statistic		0.623			0.792	

*Notes*: OR denotes odds ratio; CI denotes confidence interval; household income is standardized by household size; a non-married marital status includes never-married, separated, widowed and divorced participants; unemployed status includes students and housewives; and the *p*-value is based on the Chi-square test.

The results of the detailed decomposition are shown in Table 3. The overall composition effect contributed 4% of the gender difference in HRD, whereas the overall tendency effect accounted for 96%. As a part of the overall tendency effect, the gender-specific HRD-tendency effect explained 90% of the gender difference in HRD. The difference in HRD-tendency between men and women surveyed in 1998 was found to be the most crucial factor because it explained 11% of the gender difference in HRD. Groups whose gender differences in HRD-tendency made a positive contribution to the gender difference in HRD and their percentage contribution were the married group (8%), the 60+ group (8%), the 20-24 BMI group (2%), the 50-59 year group (2%), and the low-level stress group (2%). Conversely, those groups that showed a negative contribution to the gender difference in HRD and their percentage contributions were persons surveyed in 2005 (8%), of < 30 years (6%), those having a lower than college level of education (5%), non-married people (4%), those aged 30-39 years (2%), those surveyed in 2001 (2%), and those with manual labour jobs (1%). Among factors contributing to the composition effects, most characteristics made a small or insignificant contribution to the gender difference in HRD. Among them, the gender difference in the proportion of persons aged 60+ years and the gender difference in manual job workers each contributed to 2% of the gender difference in HRD.

**Table 3:** Detailed decomposition of the gender difference in high-risk drinking: raw estimates and percentage contributions (n = 23587)

	Composition effect (C)				Pure tendency effect (D)			
	**Raw effect**			**Contrib**	**Raw effect**			**Contrib**

	**Estimate**	**s.e**.	**p-value**	**(%)**	**Estimate**	**s.e**.	**p-value**	**(%)**

Age (years)								

< 30	-0.05	0.000	< 0.0000	-0.16	-1.930	0.001	< 0.0000	-6.48

30-39	0.10	0.000	< 0.0000	0.35	-0.698	0.001	< 0.0000	-2.34

40-49	0.12	0.000	< 0.0000	0.39	-0.192	0.001	0.1366	-0.65

50-59	-0.02	0.000	0.0291	-0.06	0.565	0.001	< 0.0000	1.90

≥ 60	0.57	0.000	< 0.0000	1.92	2.451	0.002	< 0.0000	8.23

Marital status								

Non-married	-0.12	0.001	0.0321	-0.39	-1.180	0.001	< 0.0000	-3.96

Married	-0.12	0.001	0.0300	-0.39	2.507	0.003	< 0.0000	8.42

Education								

Lower than college	-0.08	0.001	0.3231	-0.27	-1.576	0.003	< 0.0000	-5.29

College or higher	-0.08	0.001	0.3208	-0.27	0.459	0.001	< 0.0000	1.54

Occupational status								

Unemployed	0.53	0.003	0.0760	1.79	0.295	0.003	0.3615	0.99

Non-manual	-0.06	0.001	0.5735	-0.20	0.093	0.001	0.1937	0.31

Manual	0.46	0.002	0.0027	1.54	-0.441	0.002	0.0043	-1.48

Household income								

Lowest quartile	0.06	0.000	0.1098	0.20	0.105	0.002	0.4973	0.35

2nd lowest quartile	-0.01	0.000	0.0816	-0.02	-0.258	0.001	0.0502	-0.87

2nd highest quartile	0.02	0.000	0.0815	0.07	0.031	0.001	0.8051	0.11

Highest quartile	0.03	0.000	0.0705	0.10	0.116	0.001	0.3906	0.39

Body mass index								

< 20	-0.11	0.000	0.0057	-0.38	-0.177	0.002	0.4109	-0.59

20-24	0.16	0.000	< 0.0000	0.52	0.631	0.003	0.0410	2.12

25-29	0.00	0.000	0.1052	0.02	0.249	0.002	0.1041	0.84

≥ 30	-0.06	0.000	0.0149	-0.21	-0.056	0.000	0.2085	-0.19

Stress								

Very low	0.02	0.000	0.0027	0.07	0.165	0.001	0.1472	0.56

Low	-0.01	0.000	0.0004	-0.03	0.502	0.002	0.0391	1.69

High	0.00	0.000	0.0920	0.01	-0.101	0.002	0.5248	-0.34

Very high	0.01	0.000	0.0013	0.03	-0.107	0.001	0.0575	-0.36

Survey year								

1998	-0.04	0.000	< 0.0000	-0.14	3.149	0.002	< 0.0000	10.57

2001	0.00	0.000	0.0004	0.00	-0.637	0.001	< 0.0000	-2.14

2005	-0.07	0.000	< 0.0000	-0.24	-2.264	0.001	< 0.0000	-7.60

G-S tendency effect (G)	-	-	-	-	26.813	0.010	< 0.0000	90.05

Total	1.262	0.004	0.0028	4.24	28.515	0.007	< 0.0000	95.76

*Notes*: G-S tendency effect (G) denotes the gender-specific high-risk-drinking tendency effect; given the small magnitude of difference, estimates in raw effects are multiplied by 100; C+D+G equals 0.298, being consistent with the gender difference in the proportion of high-risk drinking; non-married for marital status includes never-married, separated, widowed, and divorced participants; unemployed includes students and housewives; household income is standardized by household size; s.e. denotes standard error; contrb denotes contribution.

## Discussion

In many previous studies, excessive alcohol drinking decreased with age or related to the inverted U-shape [22-24]. In addition, previous studies in Russian showed that both men and women had a tendency toward lower frequency of drinking at an older age, but this was significant only for women [18]. In this study, we found a steeper decrease in HRD with aging in women than men. According to our results, the gender difference in the HRD-tendency of persons < 30 years negatively contributed 6% in the gender difference in HRD, whereas persons of 60+ years positively contributed 8%. Based on these results, anti-HRD policies need to address men and women differently in terms of age. Moreover, in respect that the young generation, especially young Korean women, have a recent tendency toward higher HRD rate due to the their greater participation in social activities and the higher rate of their alcohol drinking. It is necessary for public health authorities to develop anti-HRD policies for them, so that they can easily recognize problems due to excessive alcohol drinking and be guided into a sound and healthy drinking culture.

We found that being married contributed negatively to the likelihood of HRD in both men and women, but this was more pronounced in women than men. A Korean culture-specific reason may be that, after being married, women are typically forced to stop working or are discouraged from HRD more strongly than men because of the rigidly patrilineal kinship system influenced by Confucianism [53]. Also, unmarried persons were generally more likely to be engaged in unhealthy behaviour, such as smoking, heavy drinking and poor diet [54,55]. The results of our decomposition analyses consistently revealed that the gender difference in the HRD-tendency in married persons was one of the strongest positive contributors (8%) to the gender difference in HRD. Considering that the prevalence of HRD is very low in married women in Korea, anti-HRD policies targeting married men might be needed to reduce their HRD-tendency and narrow the gender difference in HRD.

The relationship between education level and drinking behavior was mixed. Some studies have suggested that better educated women are more likely to abuse alcohol than their less well educated counterparts [31]. Meanwhile some studies found that the level of education is generally negatively associated with heavy alcohol consumption in both men and women [22,27-30,56], a tendency we also observed. HRD decreased when both the men and women had a higher level of education, but it was significant only for women. It may be that better-educated individuals are more likely to have greater knowledge of the risks of abusing alcohol [31,57]. Our decomposition analyses showed a negative contribution of a lower-than-college-level of education on the HRD tendency and a positive contribution of college level education or higher on the HRD tendency. Considering the results shown in Tables 1 and 2 together, anti-HRD policies seem to be relatively negatively effective in the HRD-tendency of women with a lower than college level of education and men with a college level education or higher.

The relationship between occupation and drinking behaviour is inconsistent. Most previous studies have reported that being unemployed has a positive association with HRD [2,32-34]. Employed, blue-collar and manual workers have also shown some significant relationships with heavier alcohol consumption [58,59]. However, not all studies have been consistent in this finding [58,60]. We also found that manual labourers were more likely to engage in HRD than either unemployed or non-manual labourers, regardless of gender. Considering that non-manual jobs generally are positioned at a higher place in the socio-economic hierarchy than manual jobs [61], a greater ability to pay for alcohol among the non-manual laborers enables them to engage in HRD compared to the manual laborers. However, in Korea, there is little financial barrier to engaging in HRD due to the affordability of cheap, traditional alcoholic beverages. According to our decomposition analyses, the HRD-tendency difference between men and women manual workers seemed to decrease the gender difference in HRD. This result suggests that anti-HRD policies to reduce the HRD among women with manual jobs may decrease the gender difference in HRD through the reduction of HRD-tendency difference between men and women manual workers.

Most previous studies have shown that economic difficulties are positively associated with HRD [22]. In Russia, for example, economic problems had a positive relationship with HRD in men, but not in women. The Russian study explained that growing unemployment, wage reductions, and maladjustment to stress in men resulted in increasing HRD, but that the causal relationship between economic difficulties and HRD could not be explained. We found that a higher level of household income was significantly and positively associated with HRD in men, but not significantly so in women. Compared to the poor, it is possible that a greater ability to pay for cheap or expensive alcohol among the rich enables them to engage in HRD.

BMI had inconsistent associations with HRD. It was reported in several longitudinal studies that heavy drinking in adolescence could potentially lead to overweight or obesity in adulthood [62,63]. But other longitudinal studies showed that only girl groups showed potential associations of regular alcohol drinking in adolescence with adult abdominal obesity [64]. In addition, some cross-sectional studies reported that both men [65-67] and women [65,68] showed potential associations of excessive alcohol drinking with obesity. Some possible reasons of these reports on positive associations between HRD and BMI are because many studies argue that increasing energy intake due to ingestion of alcohol is itself a major risk factor of obesity, and Korean people usually consume the traditional cheap spirit "soju" with food like pork. Our decomposition analyses suggest that if the HRD-tendency of men with a BMI of 20-24 was lowered to that of similar women, the gender difference would be reduced by 2%.

Stress and associated distress are important factors in HRD [18]. Some studies report that an alcohol disorder is driven by prior episodes of depression [69,70], whereas other studies report the contrary (i.e. alcohol disorders lead to depression) [71]. Several longitudinal studies have reported that depressive symptoms predict alcohol problems more strongly for women than for men [70,72]. Together with evidence of a positive association between stress and HRD in men and women, our study shows that the positive association was much clearer in women than men, similar to the findings of Temple et al.(1991) [73]. Because HRD is often regarded as a response to stress in people's lives, this suggests that, all other things being equal, fewer means of relieving stress other than HRD are available to women than men in Korea.

Our decomposition analysis revealed that the gender difference in the HRD-tendency of persons surveyed in 1998, 2001, and 2005 contributed significantly to an 11% increase, a 2% decrease and an 8% decrease, respectively, in the gender difference in HRD. These results indicate that after adjusting for socio-economic characteristics, women and men HRD behaviour tends to converge with time. It has also been suggested that the increasing HRD behaviour among women should be considered a target for future preventative programs.

Our study provides evidence that the gender-specific HRD-tendency effect, which represents contributions due to gender difference in a gender-specific constant term, is the major contributor to the gender difference in HRD (90%). This finding is very important because, without considering the gender-specific constant term, gender differences in HRD are only explained by the differences in socio-economic characteristics and their associated likelihood of HRD. Differences in gender-specific constant terms between men and women may be related to gender-specific characteristics such as physiology or biological predispositions, gender role orientations, social norms, knowledge of the risks of abusing alcohol [9,10,14,15,18,31,57].

## Conclusion

This is a novel study that estimates quantitatively the separate contributions of sets of factors underlying composition and tendency effects to explain the gender differences in HRD. The gender-specific HRD-tendency effect explained 90% of the gender differences in HRD in Korea. This study is meaningful in that it detected factors for which political intervention might reduce the gender differences in HRD. Moreover, it has other advantages. First, the number of subjects in this study (n = 23,587) was sufficiently large enough to be representative of the national population. Second, the importance of factors influencing the gender-specific HRD tendency have been quantified separately and emphasized.

However, the study has some limitations. First, no definite causal relationship between HRD and related socio-economic factors could be established because the study was cross-sectional in design. Second, there may be inaccuracies in the raw drinking data. Respondents were likely to report the frequency or amount of their drinking inaccurately, and some responses might have been left out, which might result in underestimating or overestimating HRD. Third, some potential factors (e.g., the price of the alcoholic beverages and peer networks) influencing gender differences in HRD could not be analyzed because national surveys in Korea have no detailed information about them. Fourth, it was impossible to report the share of observed components in explaining the gender differences in HRD. A new decomposition method to calculate the predictive power should be developed. Finally, we used the decomposition methodology of Yun (2008) [48], while alternative decomposition methods [74,75] that adopted a sequential replacement approach may provide differing results.

According to our results, gender differences in HRD-tendency (or inclination) accounted for 96% of the gender difference in HRD in Korea, whereas gender differences in observed socio-economic characteristics explained just 4% of the difference. The gender difference in the gender-specific constant term accounts for 90% of the gender difference in HRD. Factors influencing the gender-specific HRD-tendency were important in explaining the gender difference in HRD. In order to explore gender differences in HRD, more attention should be paid to factors underlying the gender-specific HRD-tendency rather than conventional factors.

In Korea, its sociocultural norms and values were formed on the basis of Confucianism from the medieval days, so alcohol drinking was traditionally considered a masculine behaviour, whereas women was excluded from drinking culture due to women's chastity and their roles as nurturer of children. That is why there have been gradually expanding social and academic interests in the issue of women's alcohol drinking and the reasons of gender difference in HRD over a few recent decades where women have more positively joined social career activities. In order to investigate why Korea has showed any occurrence of gender differences in HRD according to this sociocultural context, it is necessary to make a further comprehension about sociocultural norms and values on which Korean alcohol drinking culture is based, as well as men/women differences in socioeconomic characteristics and physiological or biological predispositions which have been addressed by previous studies. Here, it also includes any understanding about traditional gender roles and their changes in Korean society. In addition, despite more positive participations of women in social career activities and more chances for them to drink alcohol, there are still gender differences in alcohol drinking culture in Korea, such as social biases against women's alcohol drinking. Thus, to effective reduce HRD in Korea, it is necessary to understand such gender differences in social norms and values, gender roles and alcohol drinking culture as men and women experience in each society, and thereby develop any gender-differentiated intervention strategies for men and women on the basis of this understanding. Furthermore, more investigation is needed to identify factors underlying the gender-specific HRD tendency.

## Competing interests

The authors declare that they have no competing interests.

## Authors' contributions

WJC supervised all stages of the study, including originating the study and developing the analytic plan, and writing the article. SJL reviewed draft and provided critical feedback on the draft. SML helped to initiate the study and interpret findings, and provided feedback on article drafts. All authors read and approved the final manuscript.

## Pre-publication history

The pre-publication history for this paper can be accessed here:

http://www.biomedcentral.com/1471-2458/12/101/prepub
